# Medication Overuse Headaches among Children—The Contribution of Migraine and TTH

**DOI:** 10.3390/life13091902

**Published:** 2023-09-12

**Authors:** Jacob Genizi, Morya Shnaider, Liat Yaniv, Nogah C. Kerem, Keren Nathan, Irina Chistyakov

**Affiliations:** 1 Pediatric Department, Bnai Zion Medical Center, Haifa 3104802, Israel; morya2@gmail.com (M.S.); liat.yaniv@b-zion.org.il (L.Y.); nogah.kerem@b-zion.org.il (N.C.K.); keren.nathan@b-zion.org.il (K.N.); ira.chist@b-zion.org.il (I.C.); 2 Rappaport Faculty of Medicine, Technion, Israel Institute of Technology, Haifa 69094, Israel; 3 Adolescent Medicine Unit, Bnai Zion Medical Center, Haifa 3104802, Israel

**Keywords:** medication overuse headache, migraine, TTH, children

## Abstract

Medication overuse headaches are a frequent phenomenon observed in individuals suffering from chronic headaches. It arises due to the excessive consumption of pain-relief medications, resulting in the escalation and continuous persistence of headache symptoms. Nevertheless, the prevalence and distinctive characteristics of medication overuse headaches in the pediatric population have not been comprehensively explored. The primary objective of this research is to delineate the features of medication overuse headaches in children, particularly emphasizing the investigation of its epidemiology and the diagnostic patterns for headaches. We conducted a retrospective study and analyzed the medical records of children and adolescents who were evaluated at the outpatient pediatric headache clinic at the Bnai Zion Medical Center for headaches during the period spanning 2007 to 2017. Our study encompassed a cohort of 1008 patients experiencing headaches. Among these participants, 268 individuals (26.6%) were diagnosed with migraine, 250 (24.8%) exhibited tension-type headaches (TTH), and 490 (48.6%) were classified as having undifferentiated headaches. Out of the whole group, 65 had chronic headaches: 35 (54%) with migraine, 20 (30%) with tension-type headaches (TTH), and 10 (15%) with the undifferentiated headache of childhood, with the majority (73%) being female. In summary, medication overuse headaches are a prevalent issue among children grappling with chronic headaches. Intriguingly, they appear to be more pronounced within the tension-type headache (TTH) group compared to migraine sufferers and exhibit a higher prevalence among females. This study underscores the significance of early detection and careful management of medication overuse headaches in pediatric cases, shedding light on its distinct characteristics in the realm of childhood headache disorders. Further research is warranted to elucidate the underlying factors contributing to the observed gender disparity and the distinct prevalence rates among different headache subtypes.

## 1. Introduction

Medication overuse headaches (MOH) present a complex and concerning phenomenon within the realm of chronic headache disorders [1,2]. Medication overuse headaches, also referred to as medication misuse headaches or rebound headaches, arise as a result of the chronic and excessive use of pain-relieving medications, leading to the paradoxical escalation of headache frequency and severity. This condition often occurs when individuals resort to using medications intended to alleviate pain in an excessive and regular manner, ultimately culminating in a cycle of worsening headaches. Medication overuse headaches can pose a significant challenge in the realm of headache management, as the very medications intended to provide relief can inadvertently contribute to the perpetuation of headaches. This intricate interplay between medication use and headache exacerbation underscores the need for a comprehensive understanding of medication overuse headaches and its underlying mechanism [3]. Individuals who find themselves in the throes of medication overuse headaches often face a unique conundrum. While their intention is to alleviate discomfort through medication, they inadvertently find themselves caught in a cycle, where the medications themselves become a source of headache exacerbation. Recognizing the signs and symptoms of medication overuse headaches is pivotal for healthcare providers, as prompt intervention and a tailored treatment approach can break the cycle of overuse and alleviate the burden of chronic headaches. In managing medication overuse headaches, a multi-faceted approach is essential. This encompasses not only addressing the immediate symptoms but also delving into the underlying triggers and factors that contribute to medication overuse. By addressing these components, healthcare professionals can guide patients toward more effective and sustainable headache management strategies, mitigating the impact of medication overuse headaches on their daily lives [3]. As the prevalence of medication overuse headaches continues to rise, further research and education are imperative to equip both healthcare providers and patients with the tools to recognize, address, and ultimately alleviate the challenges posed by medication overuse headaches.

While extensive research has been conducted on medication overuse headaches in adults [4], the understanding of this condition in the pediatric age group remains limited. As a result, it is crucial to investigate the underlying mechanisms, risk factors, and clinical presentations of medication overuse headaches in children to develop effective management strategies and preventive measures.

Extensive literature focusing on medication overuse headaches has been published by authors studying adult populations. However, Find et al. [5] reported that a significant proportion (64.4%) of adults experiencing medication overuse headaches began experiencing their headaches during adolescence, underscoring the critical importance of addressing medication overuse headaches in the pediatric population.

The prevalence of pediatric patients suffering from chronic migraine is estimated to be within the range of 0.79% to 2% [6]. Moreover, some researchers have published prevalence rates ranging between 20% and 50% of medication overuse headaches in pediatric patients with chronic headaches [7]. Several risk factors associated with medication overuse headaches have been identified, including low socioeconomic status, stress, sedentary lifestyle, depression, anxiety, obsessive-compulsive disorder, frequent episodic migraines or tension-type headaches, the presence of other underlying causes of body pain, and sleep disturbances [8].

Medication overuse headaches are frequently reported among migraine patients [6,9]. Constantinidis et al. [10] in their study compared migraine and tension-type headache (TTH) patients and found a similar incidence of medication overuse headaches in both groups, with 50.5% of migraine patients and 45.9% of TTH patients experiencing this condition. There have been few studies that investigated the prevalence of medication overuse headaches among children with migraines [3]. However, information on medication overuse headaches in the context of TTH is scarce.

The primary objective of our study is to delve into the world of medication overuse headaches in children, with a focus on its epidemiology, clinical characteristics, headache diagnosis, and potential contributing factors. By examining the prevalence and risk factors associated with medication overuse headaches in pediatric patients, we aim to shed light on the growing burden of this condition and provide insights into developing targeted interventions and preventive strategies.

## 2. Patients and Methods

A retrospective study, reviewing the medical records of children and adolescents who were referred by their primary physicians, for neurological assessment at the outpatient pediatric headache clinics in Bnai Zion Medical Center was conducted. The review was conducted on charts of children aged 5 to 18 years who presented with headaches between the years 2007 and 2017. Migraine, TTH, and medication overuse headaches were diagnosed according to the international headache classification IHS III [11].

The data collected were analyzed statistically using the chi-square test or Fisher’s exact test for categorical data and the independent t-test for continuous variables. Odds ratios and their 95% confidence intervals were calculated, and significance was considered at a *p*-value of less than 0.05. To identify potential predictors, multiple logistic regression analysis was performed with gender, age, and family history of migraines as the main effects. Stepwise logistic regression was used to explore two-way interactions. The statistical analysis was carried out using SPSS software version 21 (SPSS, Chicago, IL, USA), and the study received approval from the local Helsinki Committee BNZ 109-18.

## 3. Results

During the study period, our clinic examined a total of 1008 patients with headaches. Their diagnoses were as follows: 268 (26.6%) patients were diagnosed with migraine, 250 (24.8%) with tension-type headaches, and 490 (48.6%) with undifferentiated headaches. (Table 1). Out of the whole group, 65 had chronic headaches: 35 (54%) with migraine, 20 (30%) with tension-type headaches (TTH), and 10 (15%) with undifferentiated headache of childhood. Only 26 (2.6%) children fulfilled the IHS criteria [11] for medication overuse headaches. In most cases of medication overuse headaches, 73% (19 patients) were females, which is statistically significantly higher than in the whole group (73% vs. 52% *p* = 0.033). The average age in the medication overuse headaches group was 12.4 years, while the average age in the other treated headache groups was only 11 years (*p* = 0.024).

As for headache diagnosis, the majority of medication overuse headaches cases were associated with tension-type headaches, accounting for 42% (11 patients), while migraines accounted for only 23% (6 patients). TTH was the most common headache diagnosis among medication overuse headaches. It was much more common than among those not overusing medications (42% vs. 24%) (Table 2). The headache symptom duration was much longer among the medication overuse headaches group (12 months vs. 25 months, *p* = 0.04).

**Figure 1:**
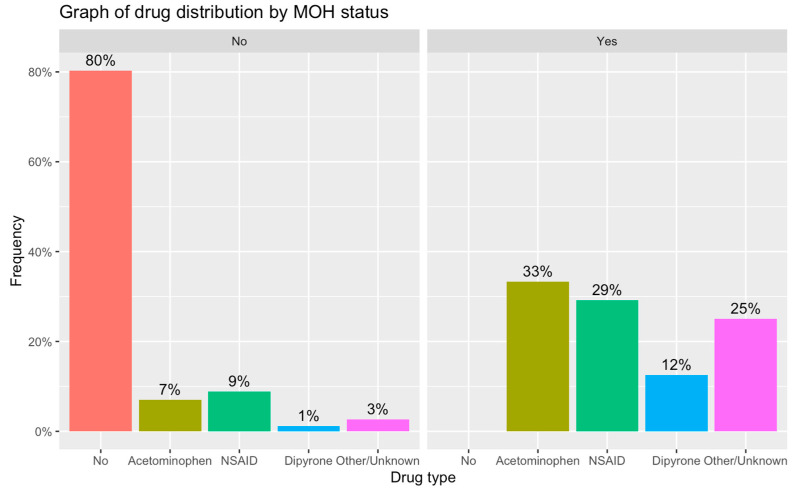
Type of analgesic use among the non-medication overuse and medication overuse headaches groups.

The two most common drugs used excessively were paracetamol (42%) and NSAIDs (38%) (Table 3). In some cases, a combination of different types of drugs was used. There was no documented usage of barbiturates, opioids, or triptans in the examined cases (Figure 1).

## 4. Discussion

In our study, we found that out of the entire group of children with headaches, 26 children (2.6%) experienced medication overuse headaches. However, among the subgroup of children with chronic headaches, the prevalence of medication overuse headaches was as high as 40%. The estimated prevalence of medication overuse headaches among adults varies significantly across different studies, ranging from 0.5% to 7.2% of the adult headache population. The prevalence of medication overuse headaches among adults with chronic headaches varies from 30% to 70% [12]. These variations can be attributed to several factors, including the country where the study was conducted, the characteristics of the study sample, the research methods employed, and the specific definition used for the condition. Loder and Scher [13] expressed their concern regarding the prevalence estimates of medication overuse headaches in adults. They noticed a significant disparity in the geographic distribution of articles on this topic. For instance, a cross-sectional study conducted in Denmark with a large adult group reported a prevalence of 1.8% [14]. On the other hand, smaller-scale studies conducted in Russia and Iran reported higher prevalence rates, with values reaching up to 7.2% [15].

Limited research has been conducted on the prevalence of medication overuse headaches among children. Soee [16] reported that the prevalence of medication overuse headaches in the pediatric headache population falls within the range of 4% to 11.76%. Similarly, Papetti [17] found a prevalence of 10.8% among pediatric patients, which is much higher than reported in adults. For children suffering from chronic headaches, some researchers have noted a higher prevalence of medication overuse headaches, with rates ranging from 20% to 50% [3]. Moreover, Heyer et al. [18] reported an even more elevated prevalence of 70% among pediatric patients experiencing post-traumatic headaches. Our hypothesis proposes that children could be more vulnerable to developing medication overuse headaches because of their potential challenges in enduring pain and their inclination to avoid taking medications when compared to adults.

In our study, as mentioned above, the numbers were closer to those reported in adults. The susceptibility of children to medication overuse headaches has given rise to a hypothesis that necessitates investigation. This proposition posits that children, due to their unique characteristics and psychological traits, could be more susceptible to developing medication overuse headaches in comparison to adults. The intricate interplay between pain endurance, medication avoidance, and the potential repercussions of these factors on headache patterns forms the core of this hypothesis.

Children inherently possess a distinct relationship with pain [19]. Their developing nervous systems and sensory perceptions could potentially influence their ability to tolerate pain and discomfort. It is plausible that children, in comparison to adults, might find it more challenging to endure and manage persistent pain, thereby leading to a heightened reliance on pain-relieving medications. This inclination towards seeking immediate relief aligns with the premise of medication overuse headaches, where excessive and chronic use of medication paradoxically exacerbates the very condition it aims to alleviate.

Moreover, children’s perception of medication could contribute to their vulnerability to medication overuse headaches. Unlike adults who may approach medications with caution or restraint, children could be less cognizant of the potential consequences of excessive medication usage. Their limited understanding of medication dynamics and long-term effects might lead them to employ pain-relief medications more liberally. This increased willingness to medicate at the first sign of discomfort could inadvertently contribute to the cycle of medication overuse and the development of medication overuse headaches.

Although these assertions present a compelling perspective, it is essential to acknowledge the complexity of the development of medication overuse headaches and the potential influence of numerous variables. Age-related psychological factors, developmental stages, family dynamics, and socio-cultural contexts can all shape children’s interactions with pain and medications, rendering the landscape intricate and multifaceted. Additionally, exploring the correlation between children’s vulnerability to medication overuse headaches and the specific types of pain-relieving medications they encounter is an avenue that merits investigation. The role of healthcare provider practices, parental guidance, and educational initiatives in shaping children’s perceptions of medication and pain management further enriches the narrative.

In conclusion, the proposed hypothesis postulating that children may be more vulnerable to developing medication overuse headaches due to their unique attributes and proclivities offers a thought-provoking perspective. Investigating this hypothesis will not only contribute to a deeper understanding of pediatric medication overuse headaches but also provide insights into potential interventions and preventive strategies to mitigate this burgeoning concern.

In our study, the majority of medication overuse headache cases were females, i.e., 73% (19 patients), which was statistically significantly higher than in the whole headache group.

Numerous previous studies have consistently shown a higher prevalence of medication overuse headaches among females, drawing attention to potential gender-specific factors influencing this condition. Yamanaka’s study [2] reported that 57.3% of medication overuse headaches patients were female, and a similar proportion of 56% was found in Papetti’s study [17]. In another study by Heyer [18], a noteworthy 63% of medication overuse headaches patients were female, and the highest proportion of 78% was reported by Munksgaard [19].

Headaches, and specifically migraines, are prevalent neurological disorders that exhibit significant gender disparities in terms of prevalence among females as opposed to men [20]. Epidemiological data consistently demonstrate a higher prevalence of headaches and migraine in females, indicating potential sex-specific susceptibility factors [21]. Hormonal fluctuations during the menstrual cycle, pregnancy, and menopause have been implicated as critical contributors to this disparity, highlighting the role of sex hormones in modulating pain pathways. Nevertheless, this pattern is still evident even among adolescents. The complexities underlying these gender differences in headaches and migraines require further investigation to optimize diagnosis, treatment, and management strategies, ultimately leading to improved healthcare outcomes for both men and women affected by these conditions. However, the prevalence of females in medication overuse headaches is even higher. These findings collectively underscore the recurrent trend of medication overuse headaches being more frequent in females across multiple studies [3], raising questions about the possible underlying mechanisms. The reasons behind this gender disparity remain subject to ongoing research and investigation, warranting further exploration to better understand the complex interplay of biological, psychological, and social factors that may contribute to the higher susceptibility of females to medication overuse headaches. Understanding these factors could lead to more tailored and effective approaches in managing and preventing medication overuse headaches in both genders.

In our study, the majority of medication overuse headaches cases were found to be associated with tension-type headaches, accounting for 42%, while migraines accounted for only 23%. Most of the existing literature on medication overuse headaches has predominantly focused on migraine patients, as evidenced by studies conducted by Munksgaard [19], Papetti [17], and Yamanaka [2]. However, a recent study by Constantinidis et al. [10] provided valuable insights by comparing medication overuse headaches incidence between migraine and TTH patients. Their results showed a similar occurrence of medication overuse headaches in both groups, with 50.5% of migraine patients and 45.9% of TTH patients experiencing this condition. This finding challenges the prevailing notion and highlights the importance of considering both headache types when studying and managing medication overuse headaches, emphasizing the need for a comprehensive understanding of this complex condition across different headache subtypes, especially among children and adolescents.

In our study, nine patients (35%) from the medication overuse headaches group had undifferentiated headaches (neither TTH nor migraine). Undifferentiated headache of childhood is a new entity not yet registered in the International Classification of Headache Disorders [11]. This term describes a headache that cannot be definitively diagnosed as either migraine or TTH, even though it exhibits features similar to those of migraines. According to recent studies [22,23,24], the occurrence of undifferentiated headaches of childhood ranges from 12% to 50%. In our study, the prevalence of medication overuse headaches among the undifferentiated headaches of the childhood group was between those of migraine (23%) and those of TTH (42%).

In our study, we made an intriguing discovery, as patients with medication overuse headaches reported experiencing headaches lasting for a significantly longer duration when compared to those without medication overuse headaches. Interestingly, this particular observation had not been documented in previous research, despite the consistent description of how medication overuse headaches can contribute to the perpetuation and chronicity of headaches. Our findings add a novel perspective to the understanding of medication overuse headaches and its potential impact on headache symptom duration, shedding light on a previously unexplored aspect of this complex condition. The prolonged headache duration in the medication overuse headaches group can have substantial implications on the overall quality of life and functioning of affected individuals. Addressing the issue of medication overuse headaches and effectively managing medication use is crucial in alleviating the burden of headache symptoms and improving the well-being of patients suffering from this challenging condition. Further research and targeted interventions are necessary to better understand the underlying mechanisms and to develop more effective strategies for preventing and managing medication overuse headaches in clinical practice.

The underlying pathophysiology of medication overuse headaches remains unclear. Some medications are more prone to trigger headaches. Notably, patients who overuse triptans for treating migraine attacks are more susceptible to developing medication overuse headaches compared to those overusing simple analgesics, as documented in previous studies [25,26]. This suggests the involvement of medication-specific mechanisms in the underlying pathophysiology of medication overuse headaches. This might be explained by the pathophysiological changes in medication overuse headaches, which involve neuronal hyperexcitability, leading to increased stimulation response and a habituation deficit. These alterations have been observed through various stimulation modes, such as sensory-evoked cortical potentials [27], the cold pressor test [28], and somatosensory-evoked potentials within both cephalic and extracephalic stimulation regions [27]. The hyperexcitability pattern in medication overuse headaches appears to depend on the specific overused drug. For instance, patients overusing triptans exhibit lower amplitudes in somatosensory-evoked potentials compared to those overusing NSAIDs [29], potentially reflecting triptan-induced changes in central serotoninergic transmission.

On the other hand, imaging studies using various modalities have demonstrated structural, functional, and metabolic alterations in the brains of patients with medication overuse headaches [30]. These changes affect different components of the central pain network, including areas responsible for sensory discriminative, cognitive, attentional, and emotional processing of pain. Notably, similar alterations have also been observed in other headache and pain disorders, suggesting shared pathophysiological mechanisms. Furthermore, genetic predisposition has been proposed as a contributing factor to the development of medication overuse headaches. Several genetic risk factors have been implicated, including the angiotensin-converting enzyme (ACE) insertion or deletion polymorphism, BDNF mutation [31], and polymorphisms in serotonin transporter genes [32]. Additionally, other genes associated with serotonergic and dopaminergic transmission, drug dependence, metabolic pathways, oxidative stress, and CGRP pathways have also been considered potential contributors to the pathophysiology of medication overuse headaches.

## 5. Limitations

Our study is a single-center cohort study. The limitation often encountered in single-center cohort studies is the potential for selection bias and limited generalizability. Our study is confined to a specific geographical location and healthcare setting, which may not adequately represent the diverse and heterogeneous population affected by medication overuse headaches across different regions and healthcare systems. Consequently, the findings derived from a single-center cohort study may not be applicable or extrapolatable to broader populations. Furthermore, the homogeneity of patient demographics, socioeconomic status, and healthcare practices within a single center can introduce a lack of diversity in the study cohort. This could result in an underrepresentation of certain groups, leading to biased results and limited external validity. Additionally, the inherent institutional practices, treatment protocols, and patient characteristics specific to the single center could introduce confounding factors that impact the study outcomes. The absence of a multicenter approach in single-center cohort studies also hampers the ability to assess variations in disease presentation, management practices, and outcomes across different healthcare settings. This limitation diminishes the robustness of the study findings and restricts the exploration of potential differences that may exist between various centers.

An additional constraint in our study arises from its retrospective nature. Retrospective studies, while valuable in certain research contexts, possess inherent limitations that warrant consideration. The reliance on existing data introduces the risk of incomplete or inaccurate records, potentially affecting the study’s reliability. The absence of direct control over data collection and variable measurement can lead to bias and confounding, compromising the internal validity of findings. Furthermore, retrospective designs are constrained by the inability to establish causal relationships, as they lack the experimental control present in prospective studies. Despite these limitations, retrospective studies remain a useful tool for generating hypotheses and exploring associations, provided their constraints are acknowledged and appropriately addressed.

## 6. Conclusions

Medication overuse headaches (MOH) represent a common issue observed in children suffering from chronic headaches. Within the realm of tension-type headaches (TTH), medication overuse headaches are even more widespread than among migraine patients. Interestingly, the prevalence of medication overuse headaches is higher among females, indicating potential gender-specific factors contributing to its development.

The exact mechanisms driving medication overuse headaches gender disparity remain the subject of ongoing research. Hormonal fluctuations, differences in pain processing, and individual medication use behaviors are factors that might play a role in this variation.

Addressing medication overuse headaches requires a comprehensive approach, involving healthcare providers, parents, and the affected children themselves. Open communication about medication usage and establishing appropriate pain management strategies are crucial steps in managing medication overuse headaches effectively. Encouraging non-pharmacological methods, such as lifestyle modifications, stress-reduction techniques, and cognitive-behavioral therapy, can also complement the treatment approach and reduce the reliance on medications.

In conclusion, medication overuse headaches are a concerning issue affecting children and adolescents with chronic headaches, particularly those with TTH. Understanding the gender disparities in their prevalence is essential to tailor interventions effectively. By promoting responsible medication use and exploring alternative therapies, healthcare professionals can assist in managing medication overuse headaches and improving the overall well-being of affected children.

## Figures and Tables

**Table 1:** Demographic of the research group.

		Total	Migraine	TTH	Undifferentiated Type
Total cases		1008	268	250	490
Gender	Male (%)	480 (48)	148 (55.1)	102 (40.8)	230 (47.1)
	Female (%)	528 (52)	120 (44.9)	148 (59.1)	260 (53)
Average age (years)		11	11.5	11.23	10.46

**Table 2:** Headache type among the medication overuse headaches subgroup.

Characteristics	Medication Overuse Headache		*p*-Value
	No	Yes	
	*n* = 986 ^1^	*n* = 26 ^1^	
Age	10.9 (3.4)	12.4 (3.0)	0.024
Gender			0.033
M	474 (48%)	7 (27%)	
F	512 (52%)	19 (73%)	
Headache type			0.11
Migraine	262 (27%)	6 (23%)	
Tension type	241 (24%)	11 (42%)	
Undifferentiated type	483 (49%)	9 (35%)	
Symptoms duration (months)	12 (15)	25 (28)	0.04

^1^ Mean (SD); *n* (%).

**Table 3:** Analgesic use among the headache group.

Characteristics	Medication Overuse Headache		*p*-Value
	No *n* = 986 ^1^	Yes *n* = 26	
Drug			<0.001
No	774 (80%)	0 (0%)	
Acetaminophen	68 (7.1%)	8 (33%)	
NSAID	85 (8.8%)	7 (29%)	
Dipyrone	11 (1.1%)	3 (12%)	
Other/Unknown	26 (2.7%)	6 (25%)	

^1^ Mean (SD); *n* (%).

## Data Availability

Data are unavailable due to privacy restrictions.
